# The Long Term Effect of Elective Colpoperineoplasty on Sexual Function in the Reproductive Aged Women in Iran

**DOI:** 10.1155/2014/912786

**Published:** 2014-10-28

**Authors:** Safieh Jamali, Parvin Abedi, Athar Rasekh, Razieh Mohammadjafari

**Affiliations:** ^1^Department of Midwifery, Nursing & Midwifery School, Jahrom University of Medical Sciences, Jahrom 74179 78681, Iran; ^2^Community Nutrition, Department of Midwifery, Reproductive Health Promotion Research Center, Ahvaz Jundishapur University of Medical Sciences, Ahvaz 61357 15794, Iran; ^3^OB & GYN Department, Jahrom University of Medical Sciences, Jahrom 74179 78681, Iran; ^4^OB & GYN Department, Ahvaz Jundishapur University of Medical Sciences, Ahvaz 61357 15794, Iran

## Abstract

*Objective*. Many women are suffering from sexual dysfunction followed by vaginal laxity in their reproductive age. The aim of this study was to evaluate the long term effect of colpoperineoplasty on sexual function in Iranian reproductive aged women. *Methods*. This was a prospective observational study in which 79 women with vaginal laxity who were candidate for selective colpoperineoplasty in Jahrom, Iran, were recruited. Data on sexual function was collected via the Female Sexual Function (FSFI) questionnaire preoperatively, six months and 18 months after colpoperineoplasty. The paired *t*-test, Wilcoxon, Mann-Whitney, and Repeated Measure test were utilized for statistical purposes. *Results*. Seventy-six women completed the study by 18 months. The mean FSFI score changed from 24.19 ± 3.09 in baseline to 26.92 ± 3.41 after six months (*P* < 0.001); however dyspareunia and vaginal dryness were increased significantly. After 18 months all areas of sexual function including pain and lubrication improved significantly compared to the 6th month (*P* < 0.001). Sexual satisfaction was increased significantly six and 18 months after surgery (*P* < 0.001), and the total score of sexual function increased to 32.61 ± 1.32 after 18 months (*P* < 0.001). *Conclusion*. The long term effect of colpoperineoplasty in women who suffer from vaginal laxity is promising. It seems that patient's dissatisfaction of sexual function can be a basis for colpoperineoplasty.

## 1. Introduction

Many women in reproductive age are suffering from sexual dysfunction followed by vaginal laxity. Normal vaginal birth and the higher number of vaginal deliveries are predisposing factors for this matter as well as pelvis organ prolapse [1]. Other predisposing factors include obesity, strenuous physical activity, and chronic coughs due to the chronic respiratory disease [2]. An epidemiologic study by Olsen et al. on 149 and 554 women aged 20 years or older showed that 11.1% of these women had life-time risk of undergoing a surgery due to the pelvic prolapse or incontinency by the age of 80 [3].

There is lack of standard practice in facing patients who are suffering from vaginal laxity. There is no standard method for evaluation of vaginal laxity before and after vaginal surgery and on the other hand the patient's sexual satisfaction is very important and should be considered carefully [4].

Cosmetic surgeries in female genitals have become popular in the recent years. Much publicity about this type of surgeries is performed by surgeons; however, some professionals are questioning the quality of these surgeries. Although many surgeons claim that results of these types of surgeries are very good, there is insufficient evidence to substantiate the claim [5]. The vaginal laxity has a close relationship with sexual dysfunction. Some surgical procedures such as posterior colpoperineorrhaphy, colpoperineoplasty, or perineorrhaphy can be useful to solve this problem [6]. In a study conducted by Goodman et al., results showed that 90% of women underwent aesthetic surgeries because they wanted to obtain more subjective pleasure regarding their sexual relationship without any real reason for surgery [7].

A study conducted by Pardo et al. on 53 women who underwent colpoperineorrhaphy because of wide vagina showed that after six months, 94% of women experienced tighter vagina and they could achieve orgasm and only two patients regretted the surgery [8].

In a retrospective observational study to assess the effect of fascial posterior colpoperineorrhaphy over the five-year period, results showed that vaginal pain, dyspareunia, and vaginal laxity were all significantly reduced; however, there was no significant difference in sexual activity [9]. In a review study by Goodman, results showed that after vaginal aesthetic plastic surgeries, the rate of patient's satisfaction was 90–95% and sexual satisfaction was 80–85% [10].

The long term effects of colpoperineoplasty on sexual function are not fully understood. The rate of these types of surgeries is quite high in Iran, but there is lack of conclusive documents. The primary aim of this study was to evaluate the long term effect of colpoperineoplasty on sexual function among women who complained of vaginal laxity in Iran.

## 2. Materials and Methods

In this prospective observational study 86 women, who were candidates for elective colpoperineoplasty, were recruited nonrandomly for the study. The study design was approved by the Ethics Committee of Ahvaz Jundishapur University of Medical Sciences, Iran. The study started on May 2011 and was completed on October 2012 in a public hospital in Jahrom, Iran. Inclusion criteria were married women who complained of vaginal laxity and literate women who aged between 15 and 45 years. The exclusion criteria were women who had a history of urogenital infections, experienced recent stressful events in their life, suffered from chronic diseases, were under medication that affects sexual function, for example, antihypertensive drugs, cimetidine, and antidepressants, were smokers, and were pregnant women and those whose husbands had a history of sexual disorders. A written informed consent was obtained from each participant prior to the study and anonymity of participants was preserved. Data was selected through a sociodemographic and the Female Sexual Function Index (FSFI) questionnaires, preoperative, while FSFI was recompleted six and 18 months after surgery. During the 18-month follow-up, the participants were free to contact the researcher (SJ) in case they had any question about their surgery and sexual function.

The FSFI questionnaire contained 19 questions including two questions in the libido domain, four questions in the sexual arousal area, four questions in the lubrication area, and three questions each for orgasm, sexual satisfaction, and pain. The Persian version of this questionnaire is validated by Mohammadi et al. [11]. A five-point Likert scale was used for scoring. The score of each domain was multiplied by certain factor. The factor for desire was 0.6, 0.3 for arousal and lubrication, and 0.4 for other domains. The minimum and maximum score for FSFI were two and 36, respectively.

All women were consulted by a gynecologist and also one of the researchers who is a midwife (SJ) and were given essential information about the consequences of the surgery. The chief complaints of all women were vaginal laxity, sexual dissatisfaction, and lack of orgasm. The vaginal laxity was confirmed by a gynecologist and wide hiatus, posterior, and/or anterior wall relaxation. None of the women had obvious pelvic organ prolapse.

Colpoperineoplasty was performed under general anesthesia, by a gynecologist. The vertical incision was performed by a surgeon in the vaginal opening. The rectovaginal area was cut until the inner section of the levator ani muscle was visible. The surgeon's aid placed a finger in the rectum of the patient to be sure there is no damage to the rectum. According to the degree of vaginal laxity, the appropriate amount of tissue was removed from the vagina. Then the soft tissues and muscles especially pudendal body were tightened. In addition, the perineal body was reconstructed. The stitches started in the upper triangle of the vagina and ended at the edge of the hymen. If two fingers of surgeon were fitted in the vagina after repair, the size of vagina was considered appropriate.

All patients were carefully assessed for any signs and symptoms of bleeding and hematoma after surgery. Women were asked to return to the clinic, one week, one, two, three, six, and 18 months after surgery for checkup. Besides the examination by gynecologist, each participant was requested to complete FSFI questionnaire in the 6th and 18th months after surgery. Women could start having sexual relations at least six weeks after surgery.

The data were analyzed using SPSS version 19. The paired *t*-test was used for comparing means in the normal distributed data and the Mann-Whitney *U* test was used for data that was not normally distributed. The Wilcoxon test was utilized for comparing categorical data before and after the study. The Repeated Measure test was used for comparing sexual function score in the beginning, 6th and 18th months of the study.

## 3. Results

Seventy-nine women completed the six-month follow-up, while 76 could accomplish the study by 18 months. The mean age of women was 34.02 years and the average age of spouses was 40.6 years. Most women had a history of vaginal delivery and the average number of children was 3.18. The sociodemographic characteristics of participants are listed in Table 1.  Table 2 is demonstrating the sexual function score of participants at the beginning of the study, six and 18 months after surgery. As evident from table, all aspects of sexual function were improved significantly except for pain during intercourse and lubrication after six months (*P* < 0.001). After 18 months all areas of sexual function including pain and lubrication improved significantly compared to the 6th month of the study. There was no report of sexual dysfunction after 18 months (*P* < 0.001). The total score of sexual function was improved from 24.19 ± 3.09 before surgery to 26.92 ± 3.41 in the 6th month of study and 32.61 ± 1.32 in the 18th month of study (*P* < 0.001). The GLM repeated measure showed a significant difference in all areas of sexual function and satisfaction in two follow-up times (*P* < 0.001).

## 4. Discussion

This study was designed to evaluate the long term effects of selective colpoperineoplasty on sexual function among reproductive aged women in Iran. The short term effect of colpoperineoplasty on sexual function has been published earlier [12]. The results of this study showed that all aspects of sexual function were improved after six months except for dyspareunia and lubrication. However after 18 months the scores of pain and lubrication also were improved significantly and none of the women had sexual dysfunction.

After colpoperineorrhaphy, dyspareunia may happen due to the narrowing of the vagina [13]. However, sexual dysfunction and dissatisfaction are not entirely related to the vaginal narrowing following vaginal surgery. Vaginal nerve supply is focused on the anterior and posterior aspects of the vaginal wall [14] and mostly dyspareunia results from damage to the vaginal nerves. Decreased blood flow of vagina and genital following vaginal surgery may decrease pelvic blood flow and vaginal fibrosis and in turn result in vaginal dryness and dyspareunia [15].

A study was conducted by Moore et al. on 78 women who complained from vaginal laxity and decreased sensation with intercourse and underwent vaginal rejuvenation/vaginoplasty procedure. Results showed that after six-month follow-up, all sexual function's area significantly improved except for desire, pain, and partner premature ejaculation [16]. Our study's results after six-month follow-up are not consistent with Moore et al.'s study, since dyspareunia increased in our patients, while our results are similar to Moore et al. after 18-month follow-up. This dissimilarity may be due to the fact that almost one-third of Moore et al.'s patients had prolapse and underwent anterior-posterior repair simultaneously, while none of the women in our study underwent anterior repair. A study on 49 women who underwent vaginal repair surgery showed that after six-month follow-up, 25% of patients were concerned about vaginal pain during intercourse [17]. The results of present study are similar to Pauls et al.'s study, where after six months the rate of dyspareunia increased by almost 50% [18]. Porter et al. reported that posterior colporrhaphy alone or with other vaginal surgeries does not adversely affect sexual function and in fact it may aid in the resumption of sexual activity and significantly improving quality of life and social aspects of daily living.

In the Porter et al.'s study, dyspareunia significantly improved or was cured following surgery in 73% of 125 patients, while it worsened in 19% of patients and did not change in three. There was no change in vaginal dryness, orgasm ability, sexual desire, sexual frequency, or sexual satisfaction. Porter et al. believed that the defect of specific repair without levator ani manipulation appears to improve sexual function [19]. Our results are in line with the Porter et al.'s study, except that in our study after six months almost half of the women had sexual dysfunction and after 18 months this rate was zero.

In a study by Robinson et al., in which 34 women underwent posterior colpoperineorrhaphy and followed 41 months, the results showed that vaginal pain, dyspareunia, and vaginal laxity were all significantly reduced [20]. Our results in long term follow-up after surgery are similar to Robinson et al.

Results of multi-armed clinical trial by Barber et al. on 343 women older than 45 years with advanced prolapse or urinary incontinence showed that women reported fewer problems with their sexual relationship after surgery compared to the baseline, but overall sexual satisfaction did not change. In our study the sexual function and satisfaction were improved significantly after 18 months. One reason for this dissimilarity between our study and Barber et al. is that our participants were younger (34.02 ± 5.3 years) than that in the Barber et al.'s study (>45 years) [21].

## 5. Strengths and Limitations of the Study

This was the first time that we evaluated the long term effect of selective colpoperineoplasty on sexual function in women with vaginal laxity in Iran. Previous studies evaluated the effect of surgery on women who had other symptoms [22]. We do not have exact statistics about the rate of these surgeries in Iran, but according to the researcher's experiences, the rate of elective colpoperineoplasty due to the vaginal laxity is quite high. Women in our study were not consulted by a psychologist; they only benefited from gynecologist and also a midwife consultation.

## 6. Conclusion

The long term effect of colpoperineoplasty in women who suffer from vaginal laxity and sexual dysfunction is promising. It seems that patient's complaint of sexual dysfunction can be a basis for colpoperineoplasty.

## Figures and Tables

**Table 1:** Sociodemographic characteristics of participants.

Characteristics	*n* = 79 mean ± SD or *N* (%)
Age	34.02 ± 5.3
Age of spouse	40.6 ± 5.9
Marriage duration	15.6 ± 6.6
Number of deliveries	3.29 ± 1.71
Number of children	3.18 (1.70)
Previous mode of delivery	
Cesarean	2 (2.5)
Vaginal delivery	77 (97.5)
Vaginal delivery using forceps or vacuum	16 (21.3)
Education	
Primary	36 (45.5)
High school	29 (36.7)
Diploma^*^	11 (13.9)
University education	3 (3.7)
Spouse's education	
Primary	23 (30.4)
High school	28 (34.3)
Diploma	20 (25.3)
University education	8 (10.1)
Job	
Working	3 (3.8)
Housewife	76 (96.3)

^*^Diploma is a degree which people receive when they finish their secondary high school.

**Table 2:** Sexual function domains in study women preoperative, 6 and 18 months after colpoperineoplasty.

Sexual function domains	Before *n* = 79	After 6 months *n* = 79	After 18 months *n* = 76	*P* value
	Mean ± SD			
Libido	2.95 ± 0.84	4.76 ± 0.83	5.96 ± 0.42	*P* < 0.001
Sexual arousal	2.87 ± 0.41	4.87 ± 0.65	5.29 ± 0.43	*P* < 0.001
Orgasm	3.7 ± 1.18	5.6 ± 0.79	5.45 ± 0.51	*P* < 0.001
Lubrication	5.22 ± 0.98	2.9 ± 0.62	5.11 ± 0.46	*P* < 0.001
Sexual satisfaction	4.47 ± 0.70	5.43 ± 0.67	5.66 ± 0.42	*P* < 0.001
Pain	4.96 ± 1.28	3.81 ± 0.83	5.33 ± 0.38	*P* < 0.001
Total score of sexual function	24.19 ± 3.09	26.92 ± 3.41	32.61 ± 1.32	*P* < 0.001
Sexual dysfunction *N* (%)	58 (73.4)	37 (46.8)	0%	*P* < 0.001
